# Establishment and Characterization of a Human Small Cell Osteosarcoma Cancer Stem Cell Line: A New Possible* In Vitro* Model for Discovering Small Cell Osteosarcoma Biology

**DOI:** 10.1155/2016/3042198

**Published:** 2016-08-29

**Authors:** Gaia Palmini, Roberto Zonefrati, Cecilia Romagnoli, Alessandra Aldinucci, Carmelo Mavilia, Gigliola Leoncini, Alessandro Franchi, Rodolfo Capanna, Maria Luisa Brandi

**Affiliations:** ^1^Department of Surgery and Translational Medicine (DCMT), University of Florence, 50134 Florence, Italy; ^2^Neurofarba Department, University of Florence, 50139 Florence, Italy; ^3^Department of Traumatology and General Orthopaedics, Azienda Ospedaliera Universitaria Careggi, 50139 Florence, Italy

## Abstract

Osteosarcoma (OSA) is the most common primary malignant bone tumor, usually arising in the long bones of children and young adults. There are different subtypes of OSA, among which we find the conventional OS (also called medullary or central osteosarcoma) which has a high grade of malignancy and an incidence of 80%. There are different subtypes of high grade OS like chondroblastic, fibroblastic, osteoblastic, telangiectatic, and the small cell osteosarcoma (SCO). In this study, for the first time, we have isolated, established, and characterized a cell line of cancer stem cells (CSCs) from a human SCO. First of all, we have established a primary finite cell line of SCO, from which we have isolated the CSCs by the sphere formation assay. We have proved their* in vitro* mesenchymal and embryonic stem phenotype. Additionally, we have showed their neoplastic phenotype, since the original tumor bulk is a high grade osteosarcoma. This research demonstrates the existence of CSCs also in human primary SCO and highlights the establishment of this particular stabilized cancer stem cell line. This will represent a first step into the study of the biology of these cells to discover new molecular targets molecules for new incisive therapeutic strategies against this highly aggressive OSA.

## 1. Introduction

Inside the heterogeneous group of sarcomas we find the osteosarcoma (OSA). OSA is one of the most common primary bone tumors which occurs in childhood and in youth [1–3]. The incidence of this cancer is about 2 cases per million persons, per year [4, 5].

Principle sites, which are involved by OSA, are the metaphyseal regions of the long bones of the extremities, characterized by a rapid bone development during the adolescence. The most common sites injured are distal femur, proximal tibia, and proximal humerus [6–9]. Nowadays, despite the fact that multimodality treatment approach has increased the survival rate from 50% in adults to 70% in children, there is always a large proportion which suffers from recurrences and dissemination of the primary tumor. Unfortunately, the survival rate for these people and for whom present micrometastases at the moment of the diagnosis remains poor (<20%) [10–12].

The World Health Organization has classified the OSA as malignant bone tumor which presents several different subtypes in relation to the histology and to the area of interest of the primary tumor bulk [13].

Between the different typologies there is also the low-grade OSA, but the major types of OSA are high grade tumors, which include the small round cell osteosarcoma (SCO). SCO is an extremely rare form of OSA with an incidence of 1.3% of all diagnosed cases for OSA [14, 15].

**Figure 1:**
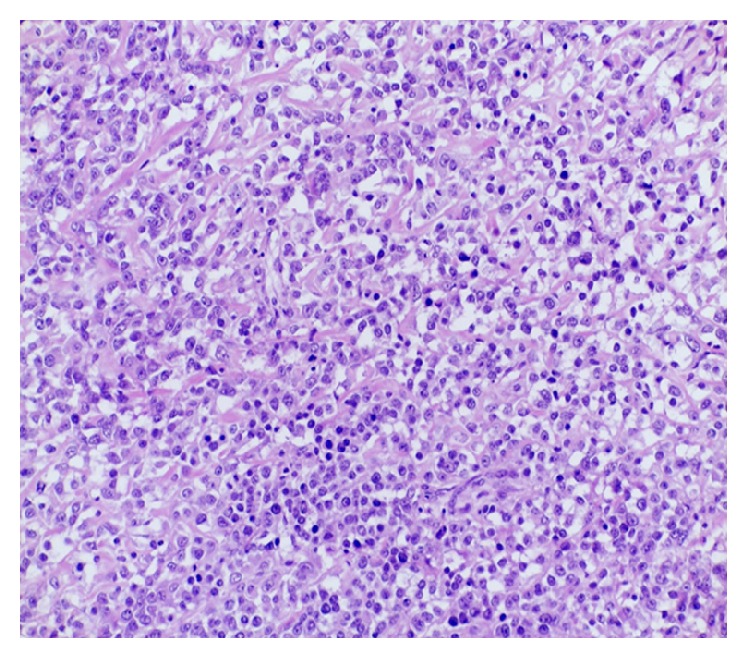
Small round cell osteosarcoma. The tumor is composed of a uniform population of round cells with focal production of osteoid matrix (H&E). Observation in brightfield. Original magnification: 10x.

Histologically the SCO is composed of small round cells with malignant phenotype, necrotic areas, and island of osteoid matrix in the stroma (Figure 1).

In any cases has been reported the production of the chondroid, too [15–19]. SCO mainly involved the femur but it can occur in all portions of the skeleton. This bone tumor can be confused by the other primary bone tumor, Ewing's Sarcoma (ES). This is possible only when the typical osteoid matrix produced by the tumor cells is not visible in the portion of biopsy. In this case, it is possible to diagnose the SCO, evaluating the immunohistochemical (IHC) expression of CD99 and SATB2, two markers of osteoblastic differentiations which are not expressed in cells of ES [4, 20–22].

The importance to make a correct diagnosis is related to the different kind of therapies which can be used against these two tumors, and which can influence the prognosis. Nowadays, as the other typologies of OSA, the therapeutic approach is multidisciplinary (surgery, chemotherapy, and radiotherapy) but unfortunately this does not permit having a good prognosis.

Additionally, it has been reported that the median survival time for a patient who did not have only the surgery but also the chemotherapy treatment is 13 years from the diagnosis of the cancer.

While this value is of 1.4 years for patients who did not have the surgery excision of the tumor bulk, in all these cases recurrence of the primary tumor and the appearance of metastases are often present and the principal site of SCO's metastases is the lung, as the other OSA [14, 23, 24].

Recent studies have also indicated the presence in bone sarcomas of a subpopulation of particular cells, called CSCs which result to be responsible to maintain and to regenerate the tumor bulk after the conventional multidisciplinary neoadjuvant therapy [25–30]. Consequently, CSCs should be present in all of the types of OSA, which are resistant to chemotherapy, like the SCO. Several studies have evaluated the presence of this subpopulation in OSA [31–34].

Since we have had the possibility of establishing a finite cell line from a human fresh biopsy of SCO, we have isolated and characterized a cell line of CSCs, in order to investigate the presence of CSCs in SCO. Hence, for the first time a CSCs line of small round cell osteosarcoma has been established.

## 2. Materials and Methods

The whole study was conducted following the approval of Florence University Hospital Ethics Committee (Rif. N. 141/12) and has therefore been performed in accordance with the ethical standards laid down in 1975 Declaration of Helsinki and its later amendments Informed Consent for tissue collection, use, and storage of the samples was obtained from the donor at AOUC.

### 2.1. Primary Osteosarcoma Cell Culture

Primary SCO cell culture has been produced in our laboratory from a fresh sample of SCO biopsy collected at the “Unità Ortopedia Oncologica e Ricostruttiva”, AOUC Careggi, Florence. The biopsy, obtained by the method of the needle aspiration, was immediately placed in a culture medium supplemented by 100 IU/mL penicillin and 100 *μ*g/mL streptomycin, pH 7.4, transported to the laboratory and processed.

The SCO primary cell culture, from the SCO tissue sample, has been set up after enzymatic treatment in Ham's F12 Coon's modification medium (Sigma-Aldrich, St. Louis, MO) with collagenase type II (Sigma-Aldrich, St. Louis, MO) at 37° and followed by mechanical dispersion. Cells were cultured as monolayer in Ham's F12 Coon's modification medium supplemented with 10% of fetal bovine serum (FBS) in a modified atmosphere of 5% CO_2_ in air at 37°C. The culture medium was changed every three days. The SCO cell culture was signed as OSA3. When cells grew to approximately 80–90% confluence, they were subcultured or harvested using Trypsin-EDTA.

### 2.2. Sphere Formation Assay

This is an* in vitro* assay used to identify and isolate cancer stem cells (CSCs) from a cancer cell line. At the 90% confluence in Ham's F12 Coon's modification medium, monolayer cells were dissociated with Trypsin-EDTA into a single-cell suspension. The cells were inoculated into sarcosphere growth medium (SGM) supplemented with 2% sterile methylcellulose (MC) at a density of 4 × 10^4^ cells/well in ultra-low attachment six-well plates (Corning Inc., Corning, NY). SGM medium consists of 2x Ham's F12 Coon's modification medium supplemented with progesterone (20 nM), putrescine 100 *μ*M, sodium selenite (30 nM), transferrin (25 *μ*g/mL), insulin (20 *μ*g/mL), the human recombinant epidermal growth factor (EGF; 10 ng/mL), and the basic fibroblast growth factor (b-FGF; 10 ng/mL) as described by Gibbs et al. [35]. All reagents were purchased by Sigma-Aldrich. Fresh aliquots of b-FGF and EGF were added every three days.

After 28 days of culture, spherical colonies formed by >50 cells were defined as “sarcospheres” and were quantitated by inverted phase contrast microscope and after that they were isolated and plated in normal attachment 60 mm diameter tissue culture plates with normal growth medium. The cells isolated from sarcospheres were signed as osteosarcoma cancer stem cells (OSA3-CSCs). When OSA3-CSCs have reached approximately 90% confluence, they were harvested and subcultured into a 100 mm diameter tissue culture plate. An additional sphere formation assay has been set up on OSA3-CSCs to investigate their ability of self-renew through secondary sphere formation. The cells isolated from this additional assay have been signed as OSA3-CSCs II.

### 2.3. Osteosarcoma Cancer Stem Cell Culture

OSA3-CSCs and the OSA3-CSCs II cell lines were cultured in a specific growth medium (GM), which is composed of Ham's F12 Coon's modification medium supplemented with 10% FBS, 100 IU/mL penicillin, 100 *μ*g/mL streptomycin, and 1 ng/mL b-FGF. The medium was refreshed twice a week and the cells were used for cryopreservation and for* in vitro* analyses to characterize their stem-like phenotype upon reaching 5 × 10^3^ cells/cm^2^.

### 2.4. Cell Line Characterization

The characterization of the cancer stem cell phenotype of the OSA3-CSCs cell line was performed by studying the doubling time, the sphere formation assay, the soft agar assay, the colony forming unit assay, the adipogenic and osteogenic differentiation, the aldehyde dehydrogenase activity analysis, the flow cytometric analyses, the immunofluorescence staining, and the gene expression. To confirm the stem phenotype of the second generation of cancer stem cells, the OSA3-CSCs II cell line has been characterized by several analyses (i.e., colony forming unit assay (CFU), differentiation stainings, immunofluorescence stainings, and gene expression analyses).

#### 2.4.1. Analysis of Cell Proliferation

OSA3-CSCs were plated in 100 mm diameter dishes at the concentration 10,000 cells/dish. After 24 hours, GM was replaced with Ham's F12 Coon's modification medium with 1.5% FBS and maintained in culture for three days. At the end, Coon's medium was replaced by Coon's medium with 10% FBS. The number of cells was evaluated at 0, 2, 4, and 6 days counting by hemocytometer chamber. The growth curve was plotted and the cell population doubling time was calculated. Each experimental point has been performed in triplicate.

#### 2.4.2. Soft Agar Colony Forming Assay for Neoplastic Transformation

The soft agar assay is an anchorage independent growth assay in soft agar, which is considered the most rigorous assay* in vitro* for detecting and proving the malignant transformation of cells. We have performed the agar soft assay on the primary cell line of SCO (OSA3), on the OSA3-CSCs, on the human osteosarcoma cell lines Saos-2 purchased from American Type Culture (ATCC, Manassas, VA), and on the mesenchymal stem cell line of preadipocytes (PA).

A 35 mm dish was coated with 1% sterile agar prepared in culture medium maintained liquid at 47°C. The dish was immediately cooled. The cell lines in growth phase were detached, suspended in medium, diluted to double the required final concentration, and maintained at 37°C. 0.33% agar was prepared in medium and maintained at 45°C.

Cell suspension was mixed with an equal volume of 0.33% agar, distributed into the agar coated dish to obtain a final concentration of 2,500 cells/dish, and immediately cooled. The cells were cultured at 37°C in humidified air with 5% CO_2_ for 4 weeks until the formation of colonies and their growth. Colonies formed per dish were observed and counted in phase contrast microscopy (Axiovert 200, ZEISS). This experiment has been performed in triplicate.

#### 2.4.3. Colony Forming Unit Assay

 OSA3-CSCs and OSA3-CSCs II lines were plated in 100 mm diameter dishes. When they had, respectively, reached 80% confluence, they were detached with Trypsin-EDTA and plated in 100 mm diameter dishes with a final concentration of 450 cells/dish. The cells were cultured in Ham's F12 Coon's modification medium with 20% FBS, 100 IU/mL penicillin, and 100 *μ*g/mL streptomycin at 37°C in humidified air with 5% CO_2_ for 4 weeks until the formation of colonies. Colonies formed per dish were stained with Toluidine Blue. The colored colonies have been counted using an inverted microscope (Axiovert 200, ZEISS). The CFU efficiency has been calculated according to the following formula: (number of colonies formed/number of cells seeded) *∗* 100. This experiment has been performed in triplicate.

#### 2.4.4. Osteogenic Differentiation

OSA3-CSCs and OSA3-CSCs II cell lines were plated on 24-well plates at a cell density of 1 × 10^4^ cells/cm^2^ in GM and grown to 80–90% confluence in each well. Afterwards, the medium was switched to osteogenic medium (OM): Ham's F12 Coon's modification medium supplemented with 10% FBS, 100 IU/mL penicillin, 100 *μ*g/mL streptomycin, 10 nM dexamethasone, 0.2 mM sodium L-ascorbyl-2-phosphate, and 10 mM *β*-glycerol phosphate. The medium was refreshed every three/four days. The osteogenic differentiation was stopped at 21 days to evaluate the osteoblastic phenotype. The main characteristics of osteoblastic phenotype are the presence of alkaline phosphatase (ALP) and of extracellular calcium mineralized deposits.

The cells were washed with DPBS (LONZA) (two times), fixed in 4% paraformaldehyde (PFA)/DPBS for 15 min, and washed with ultrapure water (three times). After that, for alkaline phosphatase (ALP) staining, the cells were washed with DPBS (two times) and stained with a specific dye mixture.

This mixture is composed of Solution A (5 mg naphthol-AS-MX phosphate sodium salt dissolved in 1 mL dimethyl sulfoxide) and Solution B (40 mg Fast Blue BB dissolved in 49 mL Tris-HCl Buffer 280 mM, pH 9.0) which are mixed together forming Solution C. 1 mL of Solution C was added for each well for 30 min at 37°C in humidified air with 5% CO_2_. ALP+ cells were stained in blue and nuclei were counterstained in red with Propidium Iodide. For mineralization staining, the cells were washed with DPBS (two times), fixed in 4% PFA/DPBS for 15 min, and washed with ultrapure water (three times). Calcium mineralized deposits were stained for 2 min with Alizarin Red S, pH 6.0, and rinsed with ultrapure water (three times). Hence, calcium mineralized deposits were stained in red-orange. ALP+ cells and calcium mineralized deposits were observed in brightfield microscopy (Axiovert 200, ZEISS).

#### 2.4.5. Adipogenic Differentiation

OSA3-CSCs and OSA3-CSCs II cell lines were cultured with a specific adipogenic medium (AM): in Ham's F12 Coon's modification medium supplemented with 10% (FBS), 100 IU/mL penicillin, 100 *μ*g/mL streptomycin and 1 *μ*M dexamethasone, 1 *μ*M bovine insulin, and 0.5 mM isobutylmethylxanthine (IBMX). The medium was refreshed twice a week. The expression of the adipogenic phenotype was evaluated on cells cultured in AM or GM for 30 days by Oil Red O staining. The colored cells were observed in brightfield microscopy (Axiovert 200, ZEISS).

#### 2.4.6. Aldehyde Dehydrogenase Activity Assay

The aldehyde dehydrogenase (ALDH) activity has been evaluated by an ALDH activity colorimetric assay kit (Sigma-Aldrich, St. Louis, MO) on the OSA3-CSCs and on a finite cell line of fibroblast, used as negative control. This kit quantifies the ALDH enzymatic activity by absorbance reading at 450 nm (VICTOR3, Perkin Elmer). The cell line was grown in 100 mm diameter dishes. When the cells had reached 100% confluence, they were detached with Trypsin-EDTA, centrifuged, resuspended in ALDH assay buffer, transferred in an ice cold 5 ml Dounce homogeniser, and disrupted. After that we have proceeded as described in the manufacturer's protocol. All tests have been done in triplicate.

#### 2.4.7. Flow Cytometry Analysis

For flow cytometry, OSA3, OSA3-CSCs, and PA were detached from tissue dishes with Trypsin-EDTA. Cells were suspended at a density of 1 × 10^5^ cells/mL in 100 *μ*L of phosphate-buffered saline with 1% bovine serum albumin and 0.1% sodium azide (Miltenyi) [36]. The antibodies were used according to manufacturer's instruction. The marked cell suspensions in 800 *μ*L were analyzed in a flow cytometer (CyFlowSpace, PARTEC).

#### 2.4.8. Immunofluorescence Staining

Immunofluorescence staining on OSA3-CSCs fixed in 4% PFA/DPBS was used to investigate the mesenchymal stem cells (MSCs) markers, using primary antibody to CD44, CD45, and CD105, and to investigate the embryonic stem cells (ESCs) and the CSCs markers, using, respectively, primary antibody to Nanog, POU5F1, SOX2, KLF4, LIN-28A, and PROM1 (CD133) and CD117. After fixation cells were permeabilized by 0.2% Triton X-100/DPBS at 37°C in humidified air with 5% CO_2_. Cells were washed three times with DPBS and were treated by RNase diluted 1/1000 with 2% BSA/DPBS at 37°C in humidified air with 5% CO_2_. After that cells were washed three times in DPBS and stained with the mouse primary antibodies (1 : 5; anti-CD44 (Invitrogen); 1 : 5; anti-CD45 (Abcam); 1 : 5; anti-CD105 (Invitrogen); 1 : 5; anti-POU5F1 (Sigma); 1 : 10; anti-PROM1 (Miltenyi)) and with the rabbit primary antibodies (1 : 5, anti-Nanog (Abcam); 1 : 5; anti-SOX2 (Abcam); 1 : 5; anti-LIN28A (Abcam); 1 : 5; anti-KLF4 (Abcam); 1 : 5; anti-CD117 (Bioss)). After incubation in a humid environment at 4°C over night the primary antibodies were removed and cells were stained with the secondary antibody (1 : 300; goat anti-Mouse Alexa Fluor 635 IgG (H+L), Life Technologies; 1 : 300; goat anti-rabbit IgG (H+L) Superclonal Secondary Antibody, Alexa Fluor 488, Invitrogen) in the dark in a humid environment at room temperature for 45 minutes. Then cells were washed several times by DPBS and counterstained for nuclei with Propidium Iodide (1 : 100 in DPBS). As negative internal control we used cells marked with only the secondary antibody. Stained cells were examined with 20x at room temperature on a Laser Scanning Confocal Microscopy (LSM 5109 Meta, ZEISS).

### 2.5. Gene Expression Analyses by Real-Time PCR

mRNA of OSA3, OSA3-CSCs, and OSA3-CSCs II was prepared by Trizol Reagent (Invitrogen, USA). Reverse transcription and Real-Time PCR (RT-PCR) analysis were carried out as described using specific primers following the manufacturer's protocol. *β*-actin was used as internal control.

First of all, we have analyzed the expression of SATB2 and of EWSR1 genes to analyze the phenotype of the primary cell line of small round cell osteosarcoma, OSA3. After that we have proceeded to characterize the CSCs lines. The expression of the ESCs and of the pluripotency marker genes (POU5F1, Nanog, SOX2, KLF4, LIN-28A, and MYC) was evaluated on OSA3, OSA3-CSCs, and OSA3-CSCs II lines.

At the same time on OSA3 and OSA3-CSCs lines have been evaluated also the expression of the cancer stem cells marker genes (prominin 1 (PROM1), aldehyde dehydrogenase 1 family, member A1 (ALDH1A1), and CD34 antigen (CD34)) and the expression of the marker genes for migration and metastasis (EZR and AXL).

All these analyses were set up on CSCs cell lines cultured in GM at the 4th passage of subculture after the isolation of the sarcospheres and on the primary cell line cultured in GM at the 1st passage of subculture.

The expression of the adipogenic phenotype in the OSA3-CSCs was evaluated on cells cultured in GM or AM for 30 days by RT-PCR analysis of the marker genes peroxisome proliferator-activated receptor (PPAR*γ*) and lipoprotein lipase (LPL). The primer sequences used for amplification of all the genes described above are listed in Table 1. Aliquots of 10 *μ*L of the amplification products were analyzed by 0.8% agarose gel electrophoresis visualized by ethidium-bromide staining.

## 3. Results

**Figure 2:**
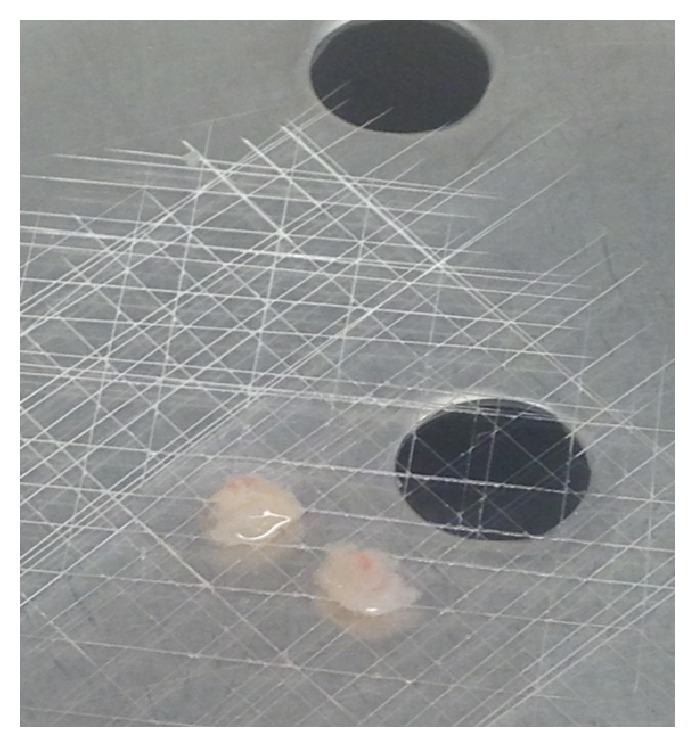
Biopsy sample of small round cell osteosarcoma. Biopsy sample of small round cell osteosarcoma by surgical resection of two small parts of the tumor.

**Figure 3:**
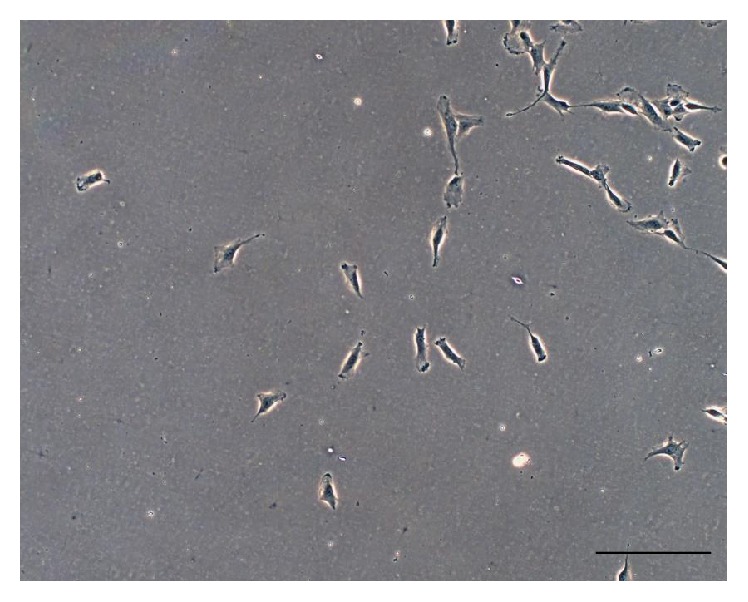
Primary cultures of small round cell osteosarcoma. Observation in phase contrast of primary cells of small round cell osteosarcoma obtained after 24 h from the mechanical disgregation of the biopsy sample. Original magnification 10x. Bar size: 100 *µ*m.

**Figure 4:**
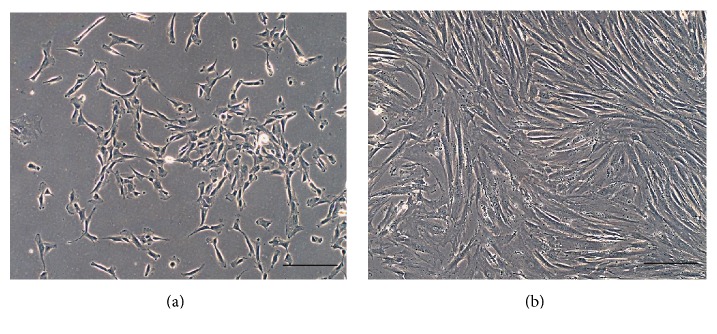
Small round cell osteosarcoma finite cell line. Observation in phase contrast of cell line of OSA3 after 15 days (a) and after 1 month (b) from mechanical disgregation.

Small round cell (SCO) sample, obtained by surgical resection of a part of the tumor (Figure 2) permits the isolation of only one osteosarcoma cell line (OSA) if treated precisely, as described before. The number of cells isolated from the small biopsy is very low (Figure 3). The output range is from 20% to 30% and it depends on the type and the dimension of the biopsy. For the primary cell line approximately one–two months is necessary to reach confluence in a 100 mm tissue dish. After this period a cell was obtained and marked as OSA3 (Figures 4(a) and 4(b)). When the cells were grown they must be subcultured to obtain sufficient number of cells to make the characterization analyses and to cryopreserve the primary cell line obtained.

**Figure 5:**
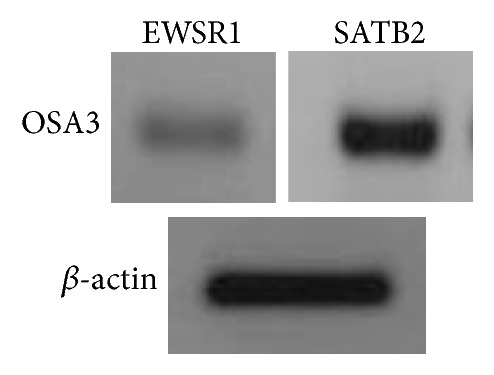
Expression of the SATB2 and EWSR1 genes in OSA3. RT-PCR show the expression of EWSR1 and of SATB2 as characterization of the OSA3 line as properly a cell line of small round cell osteosarcoma.

**Figure 6:**
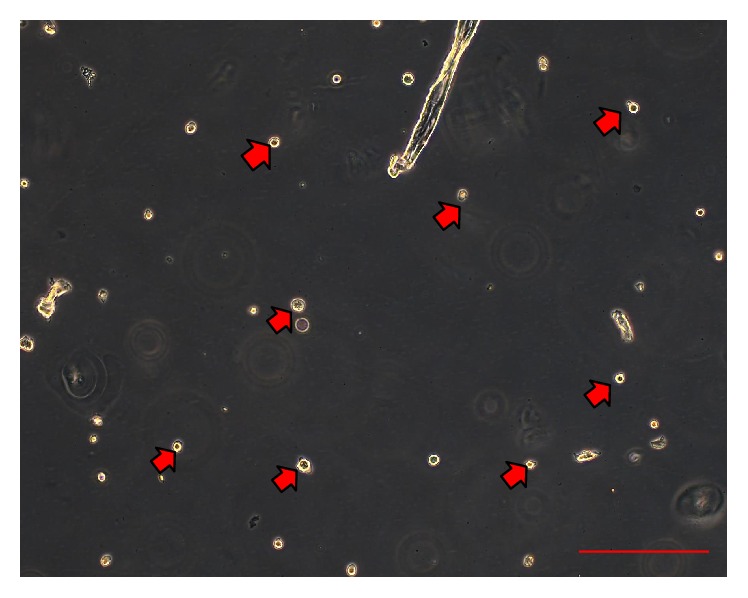
Sarcosphere assay on OSA3. After 24 h from the start of the assay, cells appear floating and isolated from each other (cells are indicated by the black/red arrows). Observation in phase contrast. Original magnification: 10x.

**Figure 7:**
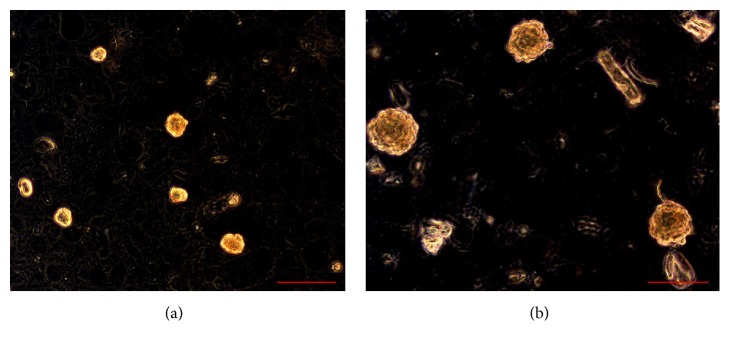
Sarcosphere assay on OSA3 at 14 and at 28 days. (a) After 14 days from the start of the assay, several small spherical colonies can be observed. The sarcospheres (indicated by the yellow arrows) appear floating in the medium or slightly settled down into the bottom of the well. (b) After 28 days big spherical colonies can be observed. Observation in phase contrast. Original magnification: 20x. Bar size: 100 *µ*m.

**Figure 8:**
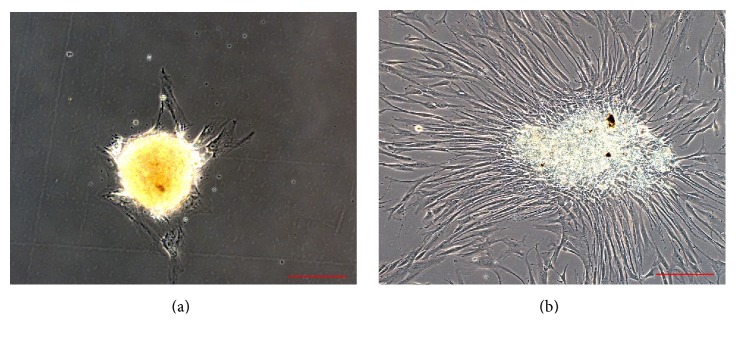
Sarcosphere after isolation. (a) Sarcospheres from OSA3 cell lines showed a beginning of adherent expansion by reintroduction and reculturing in monolayer, adherent conditions at 48 hours from the isolation. (b) At 7 days after the isolation sarcospheres showed adherent expansion by reintroduction and reculturing in monolayer, adherent conditions. Observation in phase contrast. Original magnification: 20x. Bar size: 100 *µ*m.

In relation to the characterization of OSA3 as a cell line of SCO we have evaluated the gene expression of SATB2 and of EWSR1 by RT-PCR analysis. As showed in Figure 5 OSA3 express both these genes, in particular SATB2. When the cell line at the 4th passage of subculture reached confluence, cells were detached and plated in 6-well ultra-low attachment plates for the sarcosphere assay. This particular type of plate is used because it permits us to maintain cells in a suspended state, to prevent stem cells from the attachment-mediated differentiation, to prevent the anchorage-dependent cells from dividing, and, finally, to reduce attachment to the substrate. Hence, their use permits us to create a stressful conditions which are necessary to the selection of cancer stem cells (CSCs). At 24 h after the start of the assay, cells appear isolated from one another (Figure 6). After 14 days of monitoring the progression of the assay, small spherical colonies have started to form and are visible. After 28 days, several big spherical colonies that have formed in each well can be observed and can be isolated (Figures 7(a) and 7(b)). The sarcospheres isolated were placed in normal attachment 60 mm diameter tissue dishes, under adherent conditions.  Figure 8 shows their adherent expansion in normal attachment plates.

**Figure 9:**
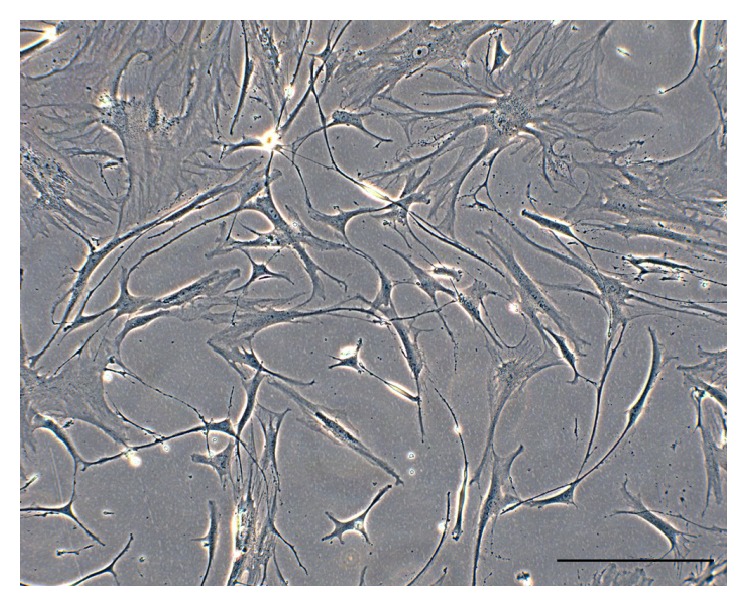
Cancer stem cell line OSA3-CSCs. Observation in phase contrast. Original magnification: 10x. Bar size: 100 *µ*m.

Cells that expand from the sarcospheres could be cancer stem cells. Consequently, after isolation, the osteosarcoma cancer stem cells (OSA-CSCs) were probably obtained and the cell line obtained was marked as OSA3-CSCs (Figure 9). As was done for the OSA3 line, it was necessary to make the OSA3-CSCs line grow and to subculture it, to cryopreserve this particular cell line, and to obtain the right number of cells to proceed with the characterization of the stem-like phenotype.

**Figure 10:**
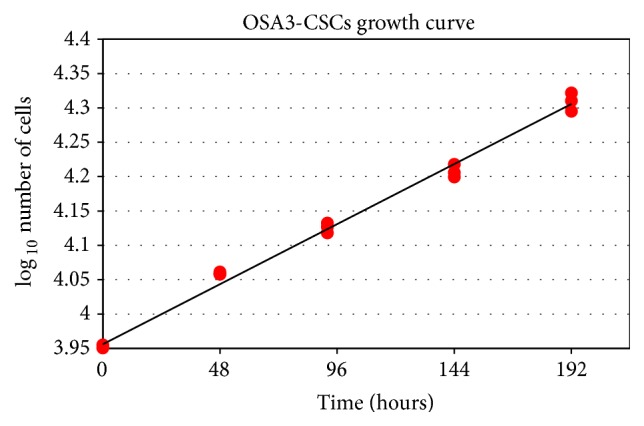
Growth curve. Graphical representation in linear regression of the kinetics of growth for OSA3-CSCs line cultured in GM (*y* = 0.0018*x* + 3.956; *R* ^2^ = 0.99). Experiment is carried out in triplicate.

All the analyses of characterization of the OSA3-CSCs cell line were performed on the 4th passage of subculture after the isolation of the sarcospheres. The OSA3-CSCs cell line showed a doubling time of 167 hours (Figure 10).

**Figure 11:**
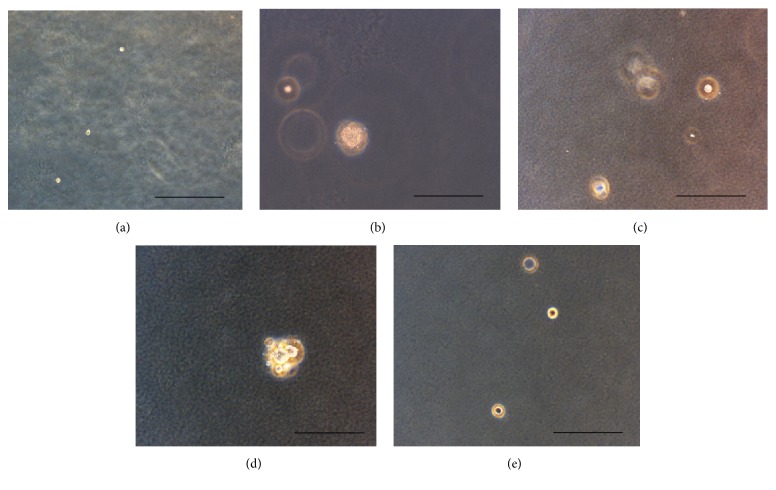
Soft agar assay on OSA3, OSA3-CSCs, Saos-2, and PA. After 24 h from the setup of the assay cells appear isolated into the agar (a). Observation in phase contrast. Original magnification 10x. While after 4 weeks spherical large colonies in OSA3-CSCs and in Saos-2 (b and d) were formed into the agar, they are absent for OSA3 (c) and PA (e). Observation in phase contrast. Original magnification 20x. Bar size: 100 *µ*m.

**Figure 12:**
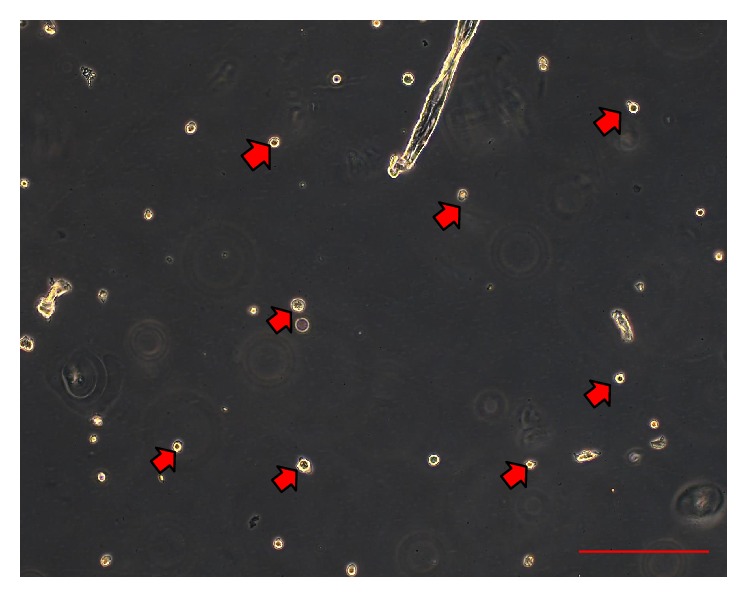
Sphere formation assay on OSA3-CSCs. After 24 h from the start of the assay, cells appear floating and isolated from each other (cells are indicated by the black/red arrows). Observation in phase contrast. Original magnification: 10x. Bar size: 100 *µ*m.

The soft agar assay has showed the capacity of the OSA3-CSCs cell line to grow in soft agar and to form large spherical colonies like the Saos-2 line, compared to the OSA3 and PA lines (Figures 11(a)–11(e)). The OSA3-CSCs showed a rate of clonogenic efficiency of 62%, similar to the rate of the SaOS-2 while the clonogenic efficiency for the OSA3 was less than 10% and was zero for PA. At the 4th passage of subcultured the OSA3-CSCs were detached and plated in ultra-low attachment plates to observe if they have maintained their capacity of self-renew (Figure 12).

**Figure 13:**
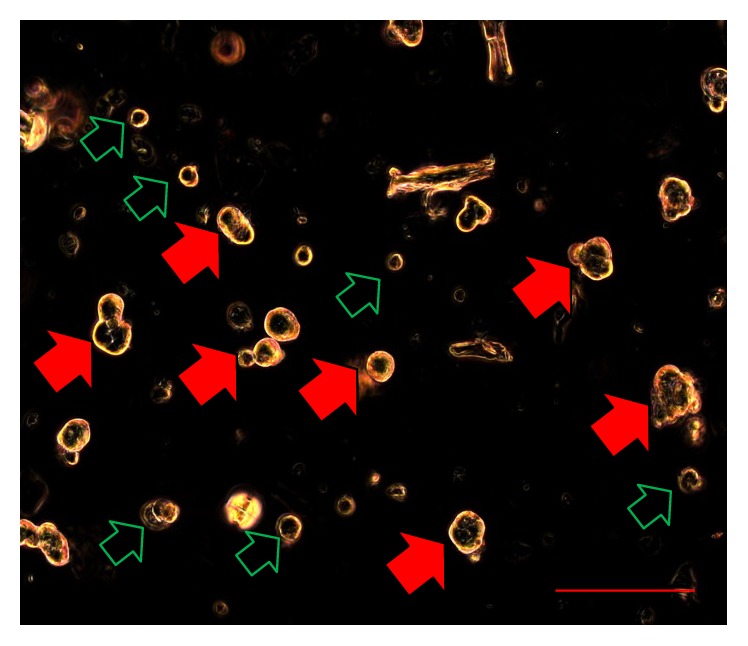
Sphere formation assay on OSA3-CSCs at 14 days. After 14 days from the start of the assay, several small spherical colonies surrounded by single cells (single cells are indicated by the black/green arrows) can be observed. The sarcospheres (some of these are indicated by the black/red arrows) appear floating in the medium observation in phase contrast. Original magnification: 20x. Bar size: 100 *µ*m.

After 14 days of the sphere formation assay, several small sarcospheres were observed (Figure 13).

**Figure 14:**
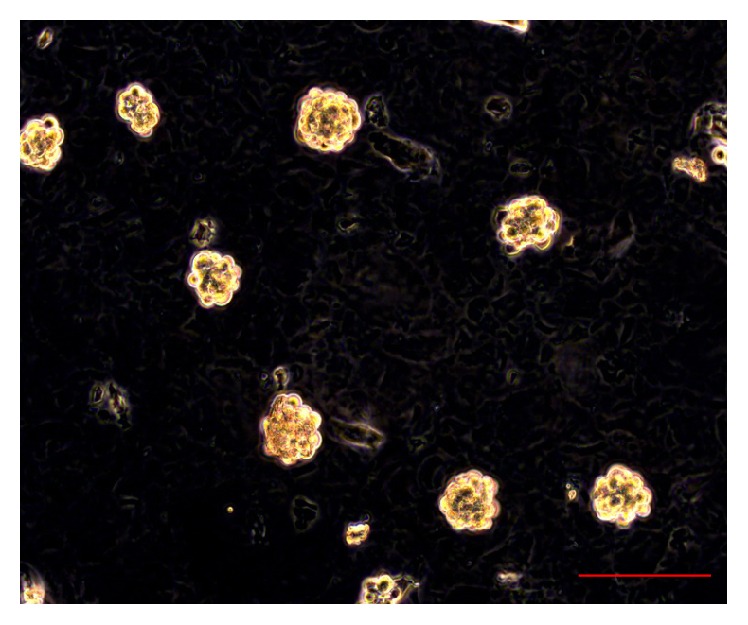
Sphere formation assay on OSA3-CSCs after 30 days. After 30 days big spherical colonies can be observed. Observation in phase contrast. Original magnification: 20x. Bar size: 100 *µ*m.

After 30 days from the start of the sphere formation assay several large sarcospheres were observed (Figure 14).

Consequently, the sarcospheres were isolated and plated in normal adherent conditions.

**Figure 15:**
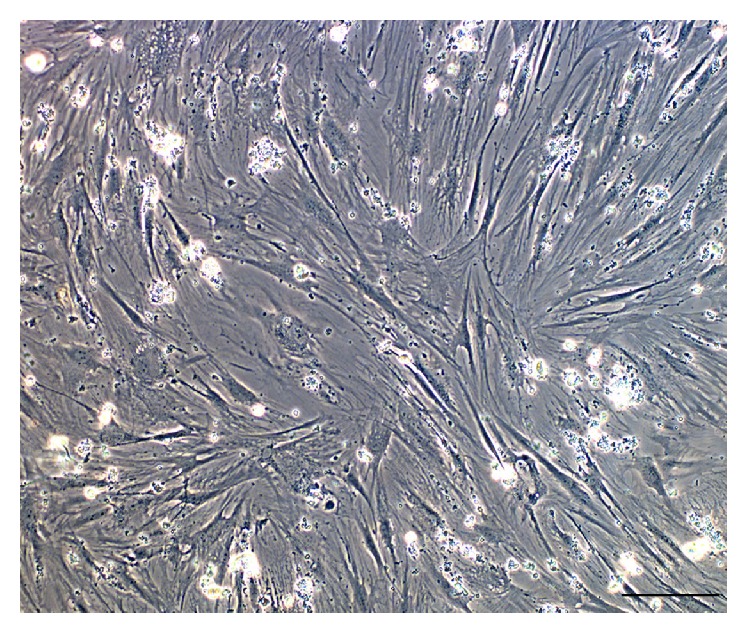
Cancer stem cell line OSA3-CSCs II. Observation in phase contrast. Original magnification: 10x. Bar size: 100 *µ*m.

The sarcospheres were isolated and the cell line was obtained in normal attachment condition after the sarcospheres isolation was indicated as the second generation of cancer stem cells from the OSA3 cell line and marked as OSA3-CSCs II (Figure 15).

**Figure 16:**
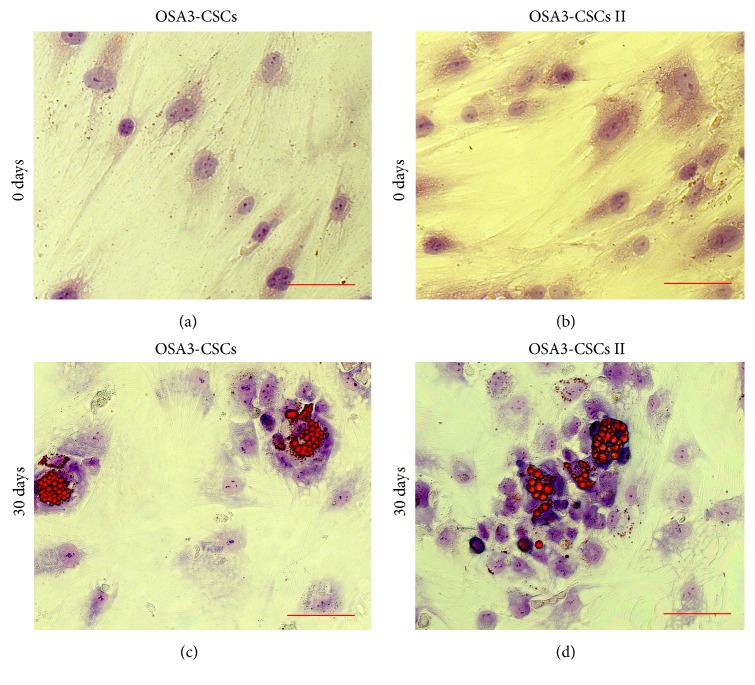
Adipogenic differentiation assay. Adipogenic differentiation at 0 days (a-b) and after 14 days (c-d) of induction by cytochemical staining with Oil Red O. In red the lipid vesicles and in blue/violet the nuclei, which have been counterstained by hematoxylin. Observation in brightfield. Original magnifications: 40x. Bar size: 100 *µ*m.

OSA3-CSCs II were made expand to evaluate if the cancer stem cell phenotype is maintained during the growth of OSA3-CSCs. Hence, OSA3-CSCs II were evaluated for their stem cell phenotype as described for their progenitor, the OSA3-CSCs. Adipogenic differentiation was not observed in both OSA3-CSCs and OSA3-CSCs II lines at 0 days, while after 30 days of adipogenic induction some cells showed the presence of intracellular vacuoles containing drops of lipids of variable shape and size (Figures 16(a)–16(d)).

**Figure 17:**
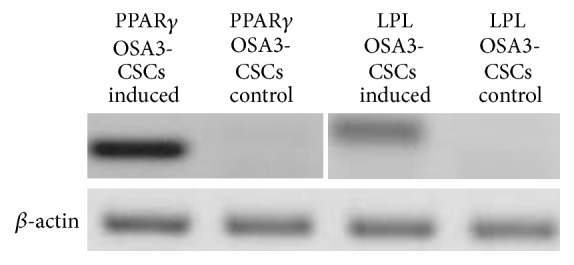
Expression of the adipogenic gene markers. RT-PCR show the expression of PPAR*γ* (left) and LPL (right), two genes involved into the adipogenic differentiation.

The adipogenic differentiation for the OSA3-CSCs line was confirmed also by RT-PCR of adipocyte-specific LPL and PPAR*γ* genes. In the absence of induction, qualitative RT-PCR showed lack of expression of both these genes, while after 30 days of induction, qualitative RT-PCR revealed a bright and intense band on agarose gel for both genes (Figure 17).

The osteogenic differentiation assay showed the capacity of both isolated OSA-CSCs lines to differentiate into osteoblast after 14 days of osteogenic induction.

**Figure 18:**
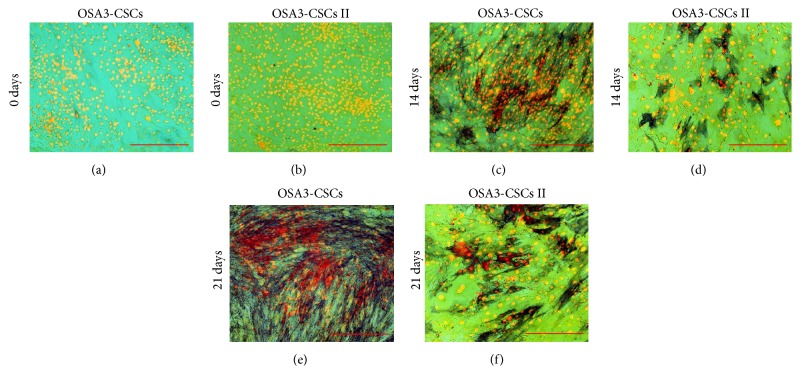
Osteogenic differentiation on OSA3-CSCs and on OSA3-CSCs II lines_ALP. Osteogenic differentiation at 0 days (a-b), after 14 days (c-d), and after 21 days of induction by a cytochemical staining for the ALP with Fast Blue BB. In blue the ALP + cells and in red the nucleus counterstained by Propidium Iodide. Composite observation in brightfield and in fluorescence. Original magnifications: 20x. Bar size: 100 *µ*m.

**Figure 19:**
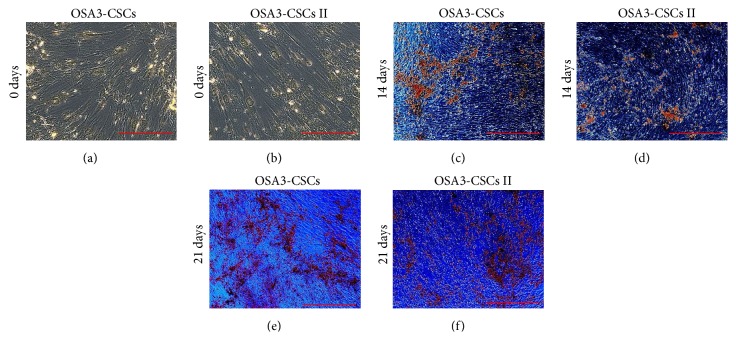
Osteogenic differentiation on OSA3-CSCs and on OSA3-CSCs II lines_HA. Osteogenic differentiation at 0 days (a-b), after 14 days (c-d), and after 21 days of induction by a cytochemical staining for the hydroxyapatite (HA) with Alizarin Red S. The cells are only contrasted in blue/grey and the grainy deposits of HA are stained in orange/red. Observation in phase contrast. Original magnifications: 20x. Bar size: 100 *µ*m.

After 21 days of induction both OSA-CSCs lines showed approximately 90% of the cell population positive to ALP and showed several calcium mineralized deposits. In both the cell lines the increase of the number of positive cells to ALP (Figures 18(a)–18(f)) and the production of calcium mineralized deposits (Figures 19(a)–19(f)) were time-dependent as observed.

**Figure 20:**
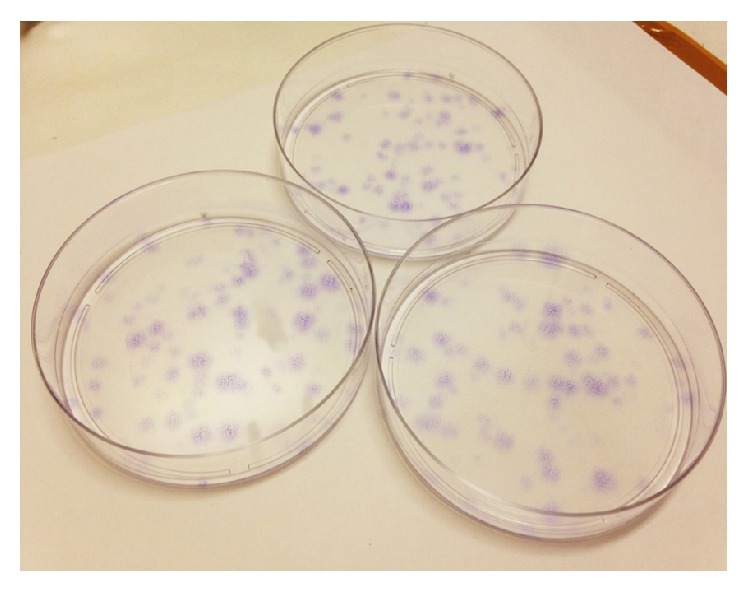
Colony forming unit assay. CFU assay on OSA3-CSCs line stained with Toluidine Blue.

Furthermore, data obtained by the CFU assay (Figure 20) have shown a good rate of clonogenic efficiency which is 27% for OSA3-CSCs and 33% for OSA3-CSCs II.

Several recent studies have shown that high levels of ALDH activity are characteristic of various types of cancer. This parameter could be used as a cancer stem cell marker and with a poor prognosis.

**Figure 21:**
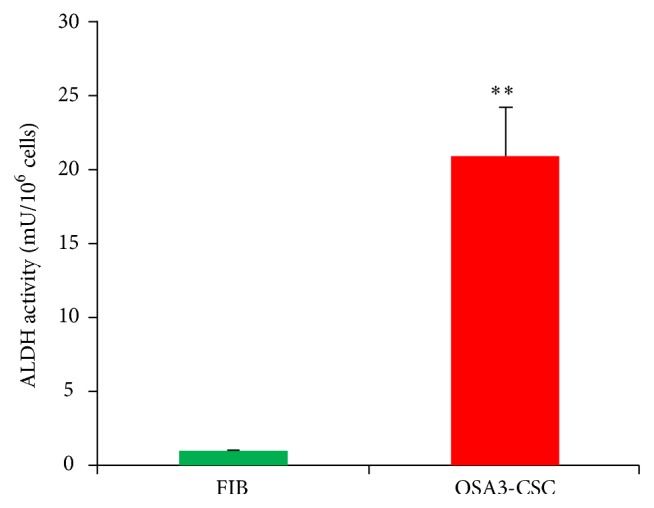
ALDH activity assay. The ALDH colorimetric assay detected high levels of ALDH activity in the cell lines of OSA3-CSCs and OSA6-CSCs while the assay detected the absence of this activity in the finite differentiated cell line of fibroblasts, FIB. Error bars: SD. ^*∗∗*^ *p* < 0.05 versus FIB.

**Figure 22:**
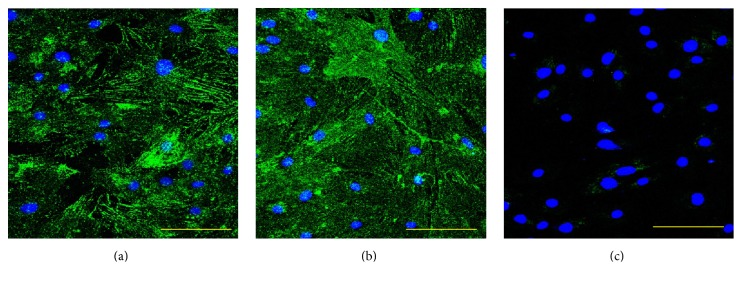
Immunofluorescence staining of CD44. Immunofluorescence staining of CD44 of the cell lines of OSA-CSCs, OSA3-CSCs (a) and OSA3-CSCs II (b). (c) Negative control. LSCM in conventional colors: green for CD44 and blue for nuclei. Original magnifications: 20x. Bar size: 100 *µ*m.

**Figure 23:**
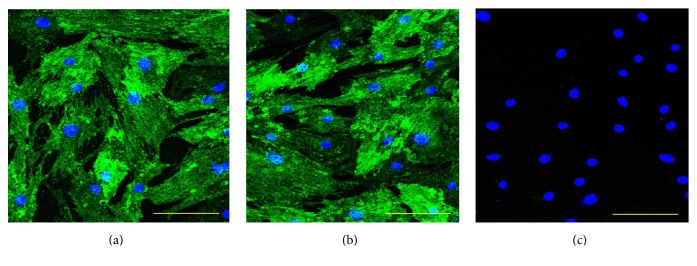
Immunofluorescence staining of CD105. Immunofluorescence staining of CD105 of the cell lines of OSA-CSCs, OSA3-CSCs (a) and OSA3-CSCs II (b). (c) Negative control. LSCM in conventional colors: green for CD105 and blue for nuclei. Original magnifications: 20x. Bar size: 100 *µ*m.

**Figure 24:**
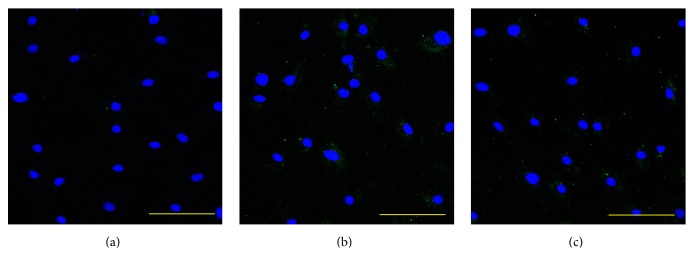
Immunofluorescence staining of CD45. Immunofluorescence staining of CD45 of the cell lines of OSA-CSCs, OSA3-CSCs (a) and OSA3-CSCs II (b). (c) Negative control. LSCM in conventional colors: green for CD45 and blue for nuclei. Original magnifications: 20x. Bar size: 100 *µ*m.

Our ALDH activity assay has showed that the OSA3-CSC line has high levels of ALDH activity (Figure 21), whereas ALDH activity was observed at the lower quantifiable limit in the fibroblast line that was used as a negative control in this assay. Both OSA3-CSCs and OSA3-CSCs II lines showed a strong positivity for the surface MSC markers (CD44 and CD105) (Figures 22(a)–22(c) and 23(a)–23(c)), while they showed a complete negativity or the hematopoietic surface marker CD45 (Figures 24(a)–24(c)). To evaluate and confirm the MSC phenotype were also performed flow cytometric analyses on OSA3, OSA3-CSCs, and a mesenchymal stem cell line of preadipocytes, marked as PA. All the results are reported in Table 2.

**Figure 25:**
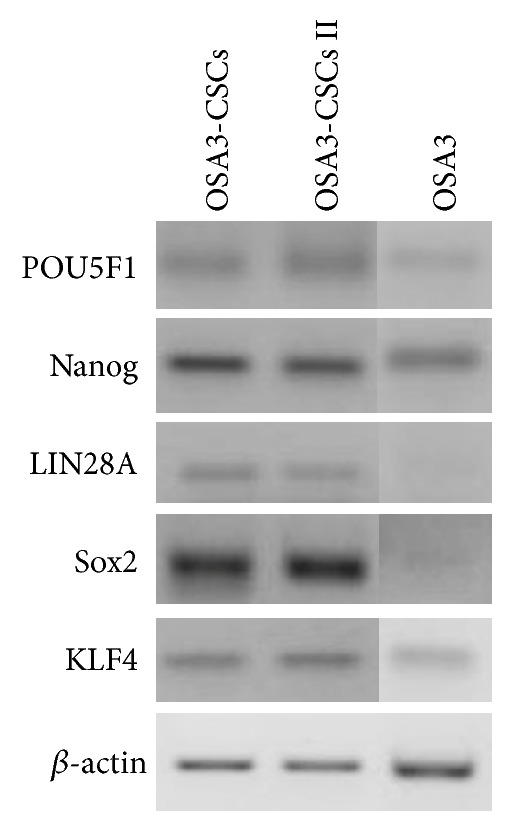
Expression of the nuclear ESC marker genes. RT-PCR show the expression of POU5F1, Nanog, LIN28A, and KLF4 in OSA3-CSCs, in OSA3-CSCs II, and in OSA3.

**Figure 26:**
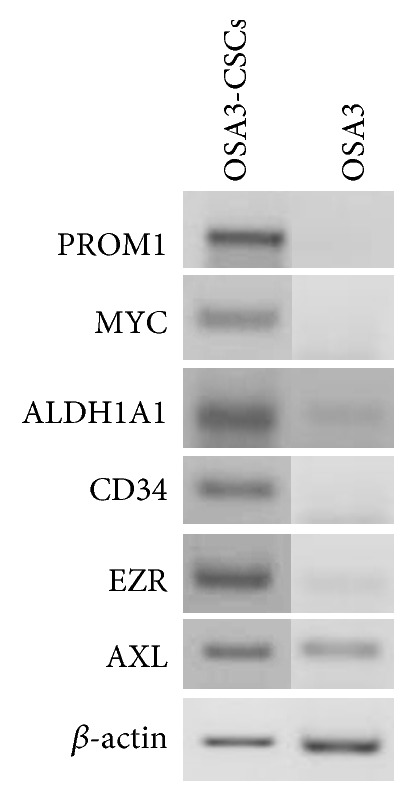
Expression of the cancer stem cells marker genes. RT-PCR show the expression of PROM1, MYC, ALDH1A1, CD34, EZR, and AXL in OSA3-CSCs and in OSA3.

By qualitative RT-PCR analysis we assessed the expression not only of the ESC marker genes (Nanog, Sox2, KLF4, LIN28A, and POU5F1) in OSA3, OSA3-CSCs, and OSA3-CSCs II (Figure 25), but also of the expression of the pluripotency marker gene, MYC, and of the other specific cancer stem cells marker genes (PROM1, ALDH1A1, CD34, EZR, and AXL) in OSA3 and in OSA3-CSC lines (Figure 26).

**Figure 27:**
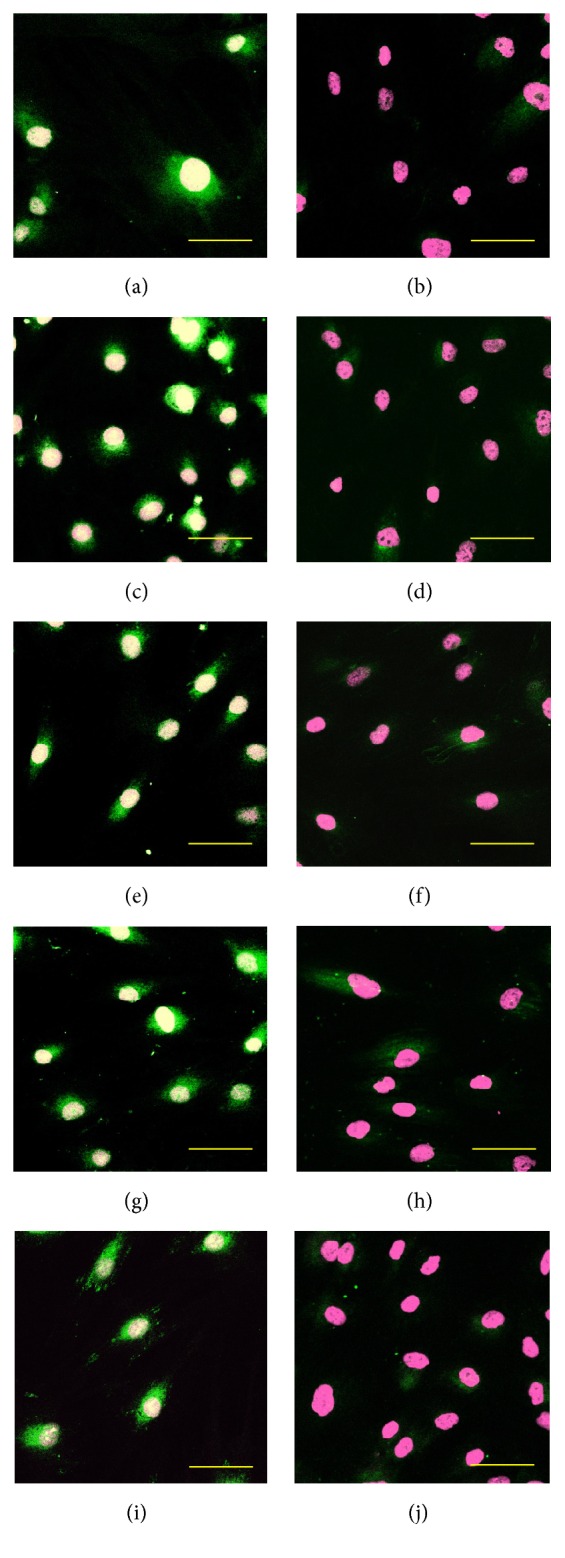
Immunofluorescence staining of ESCs markers. Immunofluorescence staining of Nanog (a), POU5F1 (c), SOX2 (e), KLF4 (g), and LIN-28A (i) of the cell line of OSA3-CSCs. (b, d, f, h, and j) Negative controls, respectively, for each marker. LSCM in conventional colors: green for ESCs and pink for nuclei. Original magnifications: 20x. Bar size: 100 *µ*m.

**Figure 28:**
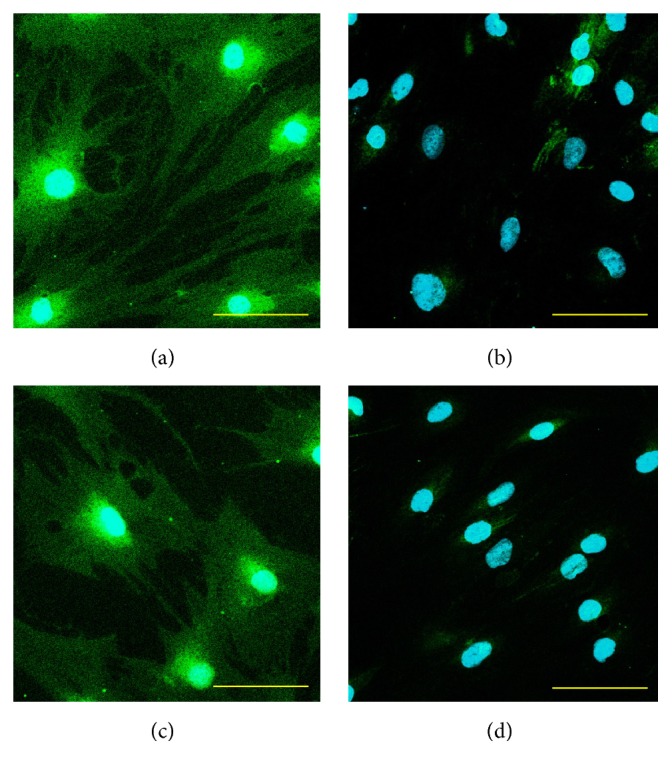
Immunofluorescence staining of CD117 and PROM1. Immunofluorescence staining of CD117 (a) and of PROM1 (c) of the cell line of OSA3-CSCs. (b and d) Negative controls, respectively, for each marker. LSCM in conventional colors: green for ESCs and sky blue for nuclei. Original magnifications: 20x. Bar size: 100 *µ*m.

We noticed that all these genes, which are characteristic of CSCs phenotype, are expressed in both the OSA3-CSCs lines, while in the OSA3 line they have resulted to be not expressed except for Nanog, KLF4, POU5F1, AXL, and ALDH1A1 genes, which have been resulted to have only a hard expression. Furthermore, by the immunofluorescence staining we have showed the presence and the nuclear localization into the cells of the ESCs marker genes (Figures 27(a)–27(j)) and of two important CSCs markers, PROM1 and CD117, which have been resulted to be localized on the surface of the cells (Figures 28(a)–28(d)).

## 4. Discussion

OSA is the most common malignant bone tumor in children and young adults [1, 2]. This is a tumor which takes origin from the connective tissue and involve the metaphyseal areas of the long bones. Despite the fact that it has a low incidence it is one of the most common primary bone cancers, thus classified by the WHO [13]. OSA tumors are a big family which includes several types of bone injuries.

One of the rarest type of high grade OSA, is the SCO [15–17]. In 1979 Sim et al. [37] were the first who for first used the term “small cell osteosarcoma” to define this rare form of OSA. Nowadays the therapeutic approach to SCO is the same used for OSA, but the prognosis for those who present this malignant tumor is always poor.

In the last decades, in the oncologic research field the new concept of CSCs has emerged, since in the 1990s they were identified and characterized into several tumors [29, 38, 39]. This is a particular cellular cluster which is located inside the differentiated tumor bulk and it is responsible to maintain the homeostasis of the tumor, to give to the malignancy the possibility of invading other tissues and, in particular, to preserve and protect the tumor cells from the chemo- and radiotherapy. All these abilities recognized to the CSCs, in relation to their stem phenotype, gave a valid explanation for why tumors, like OSA or SCO, have a poor outcome despite the neoadjuvant therapies used to treat his malignancies.

The CSCs, as mentioned before, have been just identified in several solid tumors [40–43] and, between them, there are also the bone sarcomas. The identification of these particular cells in bone sarcomas, like the OSA [32, 33], has permitted starting and studying the biology of these tumors using a new scientific approach in order to understand the mechanisms which are behind the chemoresistance of these tumors.

Consequently, to several studies which have identified CSCs in OSA, we have decided to investigate the presence of the CSCs in SCO. We have had the possibility of obtaining a fresh sample of tissue of SCO, from which we have isolated a finite cell line, marked as OSA3. After the confirmation, by the pathologist, of the tissue histotype and the establishment of the OSA3, we have evaluated the phenotype of our cell line. We have observed in OSA3 line not only a strong expression of the marker gene for the osteoblast differentiation, SATB2, but also a minimal expression of the EWSR1 gene, which is the principle marker gene for the Ewing Sarcoma [44].

Despite these particular aspects, some recent studies have reported the effective presence of EWSR1 rearrangements in few cases of SCO [45, 46] and it needs to be more investigated to understand the possible importance of this particular gene into the biology of SCO.

After all the cellular and tissue characterization of SCO, we have proceeded to investigate the stemness of the OSA3 line.

On the base of the experiments reported by Gibbs et al. [35], in relation to the possibility of isolating the CSCs by using the capacity of stem cells to survive and grow in extreme condition, like nonadherent conditions, we have isolated from the OSA3 line a cancer stem cell line, marked as OSA3-CSCs II. We have successfully established a OSA3-CSCs finite cell line and also characterized this as a real CSCs line, studying both the embryonic and the mesenchymal stem phenotype which characterized the CSCs [29].

It has been observed that OSA3-CSCs at the 4th passage of subculture were able to self-renew; in fact the sphere formation assay setup has showed their capacity to grow in nonadherent conditions and to generate several large sarcospheres, which have given origin to the “second” generation of CSCs of SCO. Our differentiation experiments have confirmed the capacity of both the CSCs lines to differentiate in the mesodermal lineages of osteoblasts and adipocytes, as can be done by the mesenchymal stem cells like the preadipocytes. The mesenchymal stem phenotype has been also confirmed by the clonogenic efficiency and in particular by the data obtained by flow cytometry on the expression of the epithelial-specific antigens CD44, CD105 and CD90, which, if they are both expressed, are recognized to be mesenchymal stem cells (MSCs) markers. Data showed that our OSA3-CSCs population isolated by the sarcospheres assay is totally positive to their presence, compared to the primary culture of SCO, which has been resulted to be very positive only to the presence of CD44. Hence, the flow cytometric analysis has showed that OSA3-CSCs line has a stemness phenotype comparable to preadipocytes. The membrane localization of the two MSCs markers is also showed by the immunofluorescence staining, which has confirmed the total absence of the other hematopoietic-specific antigen CD45. This is another important data that confirms the mesenchymal stemness of OSA3-CSCs line.

Additionally, we have evaluated and confirmed their embryonal stemness, the other aspect which characterizes the CSCs. Both the OSA3-CSCs lines have showed the expression of all the five genes which are related to the embryonic stemness (Nanog, Sox2, POU5F1, KL4, and LIN28A), while they are only a few expressed in OSA3 line. They are defined as the genes of the transcriptional core which is entirely responsible for the maintenance of the pluripotency in the embryonic cells [47–54]. So, the expression of all these genes in the CSCs has been recognized to be the base for their stem phenotype and for their ability to self-renew, not only themselves but all the tumor bulk in which these cells are included.

The embryonic stem phenotype has been also confirmed by the study of the nuclear localization of the embryonic stem cells (ESCs) markers by the immunofluorescence staining.

Since this small subpopulation has this particular property, the tumor can be resistant to the different therapies until the small cluster will not eradicated. In our study we have also characterized the malignant phenotype of the OSA3-CSCs by the analysis of the activity and the expression of aldehyde dehydrogenase 1 family, member A1 (ALDH1A1) a detoxification enzyme, which is responsible for the tumor resistance to cytotoxic chemotherapy [55]. Furthermore, only in the malignancies, we can observed high levels of ALDH1A1, and in particular in the CSCs in relation to their capacity to protect the other differentiated cancer cells.

Moreover, we have demonstrated the gene expression of another three very important genes in the characterization of the cancer stem cells. The PROM1 gene, also known as CD133+, is one of the principal markers of osteosarcoma cancer stem cells [32, 33] together with CD34, a marker of the undifferentiated state of the cells, and the ALDH1A1 genes. At the same time, we have observed the absence of these genes into the OSA3 line. Contemporary, we have also study the presence of CD133 and of CD117, other important cancer stem cells markers in osteosarcoma [55], on the surface of OSA3-CSCs, by the immunofluorescence staining.

Afterwards we have investigated also the presence of two other genes which recently have been linked to the CSCs, the EZR and the AXL genes which encode for two proteins which have been observed to be related to the malignant phenotype of a tumor [56–58]; these are involved and probably are key molecules, in the process of migration and of invasiveness operated by the CSCs. In addition to this, we have evaluated the effective capacity of the OSA3-CSCs to form colonies in agar soft assay, which is the most well established and rigorous* in vitro* assay to test the malignant transformation and the capacity to invade others tissues. Property is absent in the mesenchymal stem cells, as demonstrated by the data obtained. Hence, the data showed* in vitro* are predictive of the capacity to relapse the SCO, as described in several studies [16–20].

## 5. Conclusions

To develop an effective therapeutic strategy against OSA, and in particular against the typologies which are more aggressive and with a poor prognosis as the SCO, it is necessary not only to know the origin of this pathology, but also to understand which are the internal mechanisms responsible for the recurrence and for the invasiveness of these bone tumors. The discovery of the cluster of the cancer stem cells inside the tumors has represented the possibility of studying the mechanisms responsible for their resistance to the therapies. Consequently, our principal aim has been to demonstrate the presence of stemness into one of the rarer and aggressive histotypes of OSA.

In this research, we have collected and established a human primary cell line of SCO. Additionally, we have provided the evidence of the existence of the CSCs in SCO, too. Here, we have aimed to characterize the OSA3-CSCs line as a cellular model of human CSCs selected from primary cell line of small round cells osteosarcoma.

For the first time, we have established a CSCs line of this rare type of OSA and we have proved their* in vitro* features of CSCs.

In conclusion, our research aims to be an important first step to study* in vitro* the biology and the molecular mechanisms which are linked to the CSCs phenotype in SCO, with the principal aim to identify new molecular biomarkers cancer related. Nowadays one of the most common and famous molecular biomarkers is the large family of the small noncoding RNA, and in particular the micro RNAs (miRNAs), small interfering RNA (siRNA) and other classes of small molecules [59–62]. Several studies have demonstrated their importance in the development of the tumors and in the maintenance of their stemness [63–66], but on the other hand they have also demonstrated how these small molecules can be used as target against the maintenance of the stem phenotype of putative CSCs, representing a possible instrument against the stem cluster of a cancer [67–72]. Consequently, we have established a cellular model of CSCs from one of the rarer forms of OSA to study the presence of these targets and of their effect on his proved cancer stem phenotype with the hope to pave the way to the development of more effective focused therapeutic strategies for small cell osteosarcoma, with this still being not investigated thoroughly with an unfavorable prognosis despite the aggressive multitherapeutic approach.

## Figures and Tables

**Table 1:** Detailed list of primer sequences, with the amplicon size and annealing temperature.

Gene	Oligonucleotides	Sequence (5′-3′)	Amplicon size (bp)	*T*_*a*_ (°C)
SATB2	Forward Reverse	TGTCTATCATGTTGTGACGTTGA TCATCTCTTTGAGCAGTTCCTTTA	150	63

EWS-R1	Forward Reverse	GCAACAAAGCTATGGAACCTATG GGGCAGTGGAGTAGTAAACC	137	62

Nanog	Forward Reverse	CCCAGCTGTGTGTACTCAAT GGTTCAGGATGTTGGAGAGTT	87	60

POU5F1	Forward Reverse	GGGAGGAGCTAGGGAAAGA TCCTTCCTTAGTGAATGAAGAACT	77	60

Sox2	Forward Reverse	TGCAGTACAACTCCATGA GGACTTGACCACCGAACC	125	55

KLF4	Forward Reverse	CGGGAAGGGAGAAGACACT AGTCGCTTCATG TGGGAGA	79	60

LIN28A	Forward Reverse	CGACTGTAAGTGGTTCAAC CCTTCCATGTGCAGCTTACT	100	60

MYC	Forward Reverse	GCTGCTTAGACGCTGGATTTTT GAGTCGTAGTCGAGGCATAGT	110	63

PROM1	Forward Reverse	CCAGAAGCCGGGTCAAAAT ATTCACTCAAGGCACCATCC	127	60

ALDH1A1	Forward Reverse	TGATTCAGTGAGTGGCAAGAA CCTGCAACATCCTCCTTATCT	98	60

CD34	Forward Reverse	GTACCCTTGGAAGTACCAGCCT CAGAGGTAGATGTGAATTTGAC	100	60

EZR	Forward Reverse	GCCTTCTTGTCGATGGGTTA GCCTCTTGTCGATGGGTTTA	134	61

AXL	Forward Reverse	TTAGTGCTACGCGGAATGG CCTATGTCCATAGCACCTCG	133	60

PPAR*γ*	Forward Reverse	GTCGGTTTCAGAAATGCCTTG ATCTCCGCCAACAGCTTC	97	57

LPL	Forward Reverse	TGCATTTCAATCACAGCAGCAA TACAGGGCGGCCACAAG	101	57

**Table 2:** Expression of CD44, CD45, CD105, and CD90 in OSA3, OSA3-CSCs, and PA lines determined by flow cytometry (percentage of cells expressing the antigen).

Cell line	CD44%	CD45%	CD105%	CD90%
OSA3	99.70	0.00	0.15	14.25
OSA3-CSCs	99.74	0.00	99.52	99.39
PA	99.92	0.00	91.11	99.70
